# Male Macrophages and Fibroblasts from C57/BL6J Mice Are More Susceptible to Inflammatory Stimuli

**DOI:** 10.3389/fimmu.2021.758767

**Published:** 2021-11-18

**Authors:** Maria Luisa Barcena, Maximilian H. Niehues, Céline Christiansen, Misael Estepa, Natalie Haritonow, Amir H. Sadighi, Ursula Müller-Werdan, Yury Ladilov, Vera Regitz-Zagrosek

**Affiliations:** ^1^ Department of Geriatrics and Medical Gerontology, Charité –Universitätsmedizin Berlin, Corporate Member of Freie Universität Berlin, Humboldt-Universität zu Berlin, and Berlin Institute of Health, Berlin, Germany; ^2^ DZHK (German Centre for Cardiovascular Research), Berlin Partner Site, Berlin, Germany; ^3^ Department of Internal Medicine and Cardiology, Deutsches Herzzentrum Berlin, Berlin, Germany; ^4^ Institute for Gender in Medicine, Center for Cardiovascular Research, Charité University Hospital, Berlin, Germany; ^5^ Department of Cardiology, University Hospital Zürich, University of Zürich, Zürich, Switzerland

**Keywords:** sex differences, inflammation, bone marrow macrophages, macrophage phenotype, activated fibroblasts

## Abstract

Mounting evidence argues for the significant impact of sex in numerous cardiac pathologies, including myocarditis. Macrophage polarization and activation of cardiac fibroblasts play a key role in myocardial inflammation and remodeling. However, the role of sex in these processes is still poorly understood. In this study, we investigated sex-specific alterations in the polarization of murine bone marrow-derived macrophages (BMMs) and the polarization-related changes in fibroblast activation. Cultured male and female murine BMMs from C57/BL6J mice were polarized into M1 (LPS) and M2 (IL-4/IL-13) macrophages. Furthermore, male and female cardiac fibroblasts from C57/BL6J mice were activated with TNF-α, TGF-β, or conditioned medium from M1 BMMs. We found a significant overexpression of M1 markers (c-fos, NFκB, TNF-α, and IL-1β) and M2 markers (MCP-1 and YM1) in male but not female activated macrophages. In addition, the ROS levels were higher in M1 male BMMs, indicating a stronger polarization. Similarly, the pro-fibrotic markers TGF-β and IL-1β were expressed in activated cardiac male fibroblasts at a significantly higher level than in female fibroblasts. In conclusion, the present study provides strong evidence for the male-specific polarization of BMMs and activation of cardiac fibroblasts in an inflammatory environment. The data show an increased inflammatory response and tissue remodeling in male mice.

## Introduction

In several cardiovascular diseases, inflammatory and pro-fibrotic responses play a detrimental role (1, 2). Inflammatory processes are tightly regulated by signals that initiate and maintain inflammation and promote resolution of the inflammation (3, 4). An imbalance in these mechanisms may promote cellular and tissue damage (3).

Macrophages are a crucial part of the cardiac immune response since they are the most abundant immune cells in the heart (5). It is worth noting that cardiac macrophages interact with other cells in the heart and directly or indirectly regulate different phases of cardiac diseases: acute inflammation, immune-regulation, and resolution, as well as cardiac remodeling (6). In addition, macrophages modulate the response to various stressful conditions in the heart (7). During cardiac stress, e.g., myocardial infarction or myocarditis, the population of tissue-resident macrophages expands by recruiting from the bloodstream or local proliferation (8–10). Macrophages can be polarized into M1 macrophages, which have a pro-inflammatory signature, or into M2 macrophages, which are involved in anti-inflammatory actions, wound healing, tissue remodeling, and immune regulatory actions (11, 12). An aberrant expression of pro-inflammatory cytokines during inflammatory processes leads to the macrophage phenotype switching into a pro-inflammatory phenotype, promoting the perpetuation of the inflammation (13). Key pro-inflammatory Th1-related cytokines, e. g., interferon gamma (IFN-γ) or toll-like receptor (TLR4) signaling, induce a M1 phenotype (14), which releases pro-inflammatory mediators like tumor necrosis factor (TNF)-α, interleukin (IL)-1β, IL-6, and reactive oxygen species (ROS) (15). Macrophage differentiation into the anti-inflammatory M2 phenotype is induced by exposure to IL-4 and IL-13 (16, 17). M2-macrophages express and release anti-inflammatory molecules including IL-10, transforming growth factor beta (TGF-β), and interleukin-1 receptor antagonist (IL-1ra) (18).

Macrophages play a significant role in the cardiac remodeling of the extracellular matrix (19) by activating cardiac fibroblasts *via* TGF-β, IL-1β, and TNF-α (20, 21). The depletion of monocytes and macrophages in the myocardium following cardiac stress decreases both fibroblast activation and collagen deposition (22).

Numerous factors may modulate macrophage polarization by cytokines. In particular, macrophage polarization may be affected by sex hormones, e.g., by the main female sex hormone, estradiol (E2). Although both pro- and anti-inflammatory actions of E2 have been described (23, 24), most studies argue for the anti-inflammatory effects of estrogen receptor (ER) activation in the heart (25, 26). Several reports propose the anti-inflammatory effects of E2 are caused by the inhibition of production and the release of pro-inflammatory cytokines with a M1 signature (27). Furthermore, the ERα seems to be involved in the promotion of M2 macrophage polarization, leading to an anti-inflammatory phenotype (25, 26, 28). In keeping with that profile, E2 loss leads to the expression of pro-inflammatory cytokines e.g., IL-1β, TNF-α, and IFN-γ in humans (29). The anti-inflammatory actions of E2 in male and female peripheral blood mononuclear cells are also observed after activation with lipopolysaccharide (LPS) (25, 30). Altogether, E2 seems to suppress pro-inflammatory and promote anti-inflammatory responses. The difference in E2 blood concentration in males and females may, therefore, be responsible for the sex difference in the inflammatory response.

In this study, we investigated sex-related alterations in the polarization of murine bone marrow macrophages (BMMs) and polarization-related changes in murine fibroblast activation. The analyses revealed that male BMMs are more susceptible to LPS treatment and promote a prominent M2 phenotype. Sex differences were also found in oxidative stress, i.e., less total ROS formation in female BMMs in a pro-inflammatory environment. Moreover, we demonstrated an activation of cardiac fibroblasts with the pro-inflammatory supernatant of cultures of M1 macrophages. Finally, 17β estradiol treatment improved the pro-inflammatory phenotype in male BMMs.

## Material and Methods

### Animals

Young age-matched male and female C57/BL6J mice (n= 18) (Forschungseinrichtungen für Experimentelle Medizin (FEM), Charité -Universitätsmedizin Berlin) were euthanized and heart, femur, and tibia were collected in ice-cold DPBS (Gibco, Germany) for further processing. A 12 h/12 h light and dark cycle was applied. Water and food were provided *ad libitum*. All experimental procedures were performed according to the established guidelines for the care and handling of laboratory animals and were approved by the Animal Care Committee of the Senate of Berlin, Germany (Approval number: T0333/08).

### Cell Culture

#### Isolation and Cultivation of Murine Bone Marrow-Derived Macrophages

Bone marrow cells were collected by flushing the femur and tibia in DMEM (phenol red) (Gibco, Germany), 10% fetal bovine serum (FBS) (Biochrom, Germany), 1 mmol/l penicillin/streptomycin (Biochrom, Germany), and 1 mmol/l sodium pyruvate (Sigma, Germany) using a 20-gauge needle and were passed through a 70 µm cell strainer. Cells were cultivated for ten days (10% CO_2_ and 37°C) in DMEM (phenol red), 0.05 mmol/l β-mercaptoethanol (Sigma-Aldrich, Germany), 1% non-essential amino acids (Thermo Scientific, Germany), 1 mM penicillin/streptomycin, 1 mM sodium pyruvate, 20% donor horse serum (Sigma-Aldrich, Germany), 10% FBS (Biochrom, Germany), and 20% L929-conditioned medium.

#### Isolation and Cultivation of Murine Cardiac Fibroblasts

Hearts were cut into small pieces and digested 5 times by incubation in a collagenase/dispase buffer for two minutes at 37°C (31). The supernatant was carefully removed and diluted with ice cold growth medium (DMEM with phenol red, 10% fetal bovine serum (FBS), 1 mmol/l glutamine, 1 mmol/l penicillin/streptomycin, 1 mmol/l sodium pyruvate) and centrifuged at 1200 rpm for 5 min at 4°C. Cells were cultivated in fibroblast growth medium (DMEM, 10% FBS, 1 mmol/l penicillin/streptomycin, 1 mmol/l sodium pyruvate, and 1 mmol/l glutamine) until 80% confluence.

### Macrophage Polarization

BMMs were polarized into M1 macrophages with 10 ng/ml LPS (Sigma-Aldrich, Germany) and into M2 macrophages with 10 ng/ml recombinant mouse IL-4 (PeproTech, Germany) and 10 ng/ml recombinant mouse IL-13 (PeproTech, Germany) for 24 h.

### Fibroblast Activation

Fibroblasts were activated using 20 ng/ml TNF-α (32) (PeproTech, Germany), 10 ng/ml TGF-β (PeproTech, Germany) (33) or 10 ng/ml LPS (Sigma, Germany) for 24 h in fibroblast starvation medium (with 2.5% charcoal-stripped FCS, Biochrom, Germany).

### Activation of ERs

Cells were starved with a phenol free medium and 2.5% charcoal-stripped FCS (Biochrom, Germany) for 24 h prior to E2 treatment. After starvation, cells were treated with 10 nmol/l water soluble E2 (Sigma-Aldrich, Germany) or with 10 nmol/l dextrin (Sigma-Aldrich, Germany) as vehicle for 24 h.

### Treatment With Conditioned Medium From M1-BMMs

Mice cardiac fibroblasts were cultivated with mixture (1:1) of the fibroblast-starvation medium and conditioned medium from M1-BMMs for 24 h. To produce the conditioned medium from M1-BMMs, BMMs were treated with 10 ng/ml LPS (Sigma-Aldrich, Germany) for 24h. The cell culture medium was collected and centrifuged at 1200 rpm for 5 min at 4°C. The supernatant was stored at -80°C.

### Flow Cytometry

**Figure 1:**
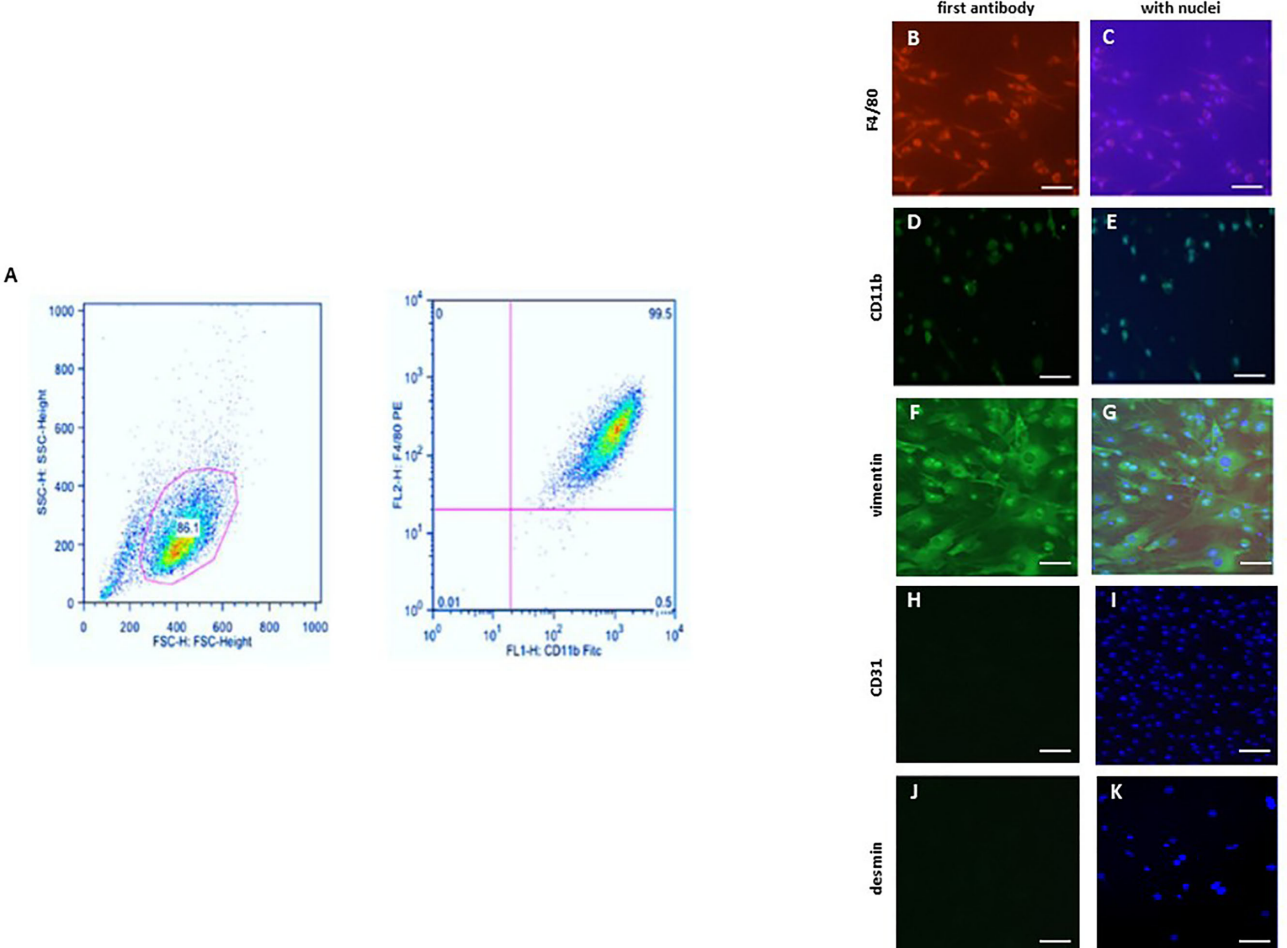
Characterization of bone marrow derived macrophages (BMMs) and cardiac fibroblasts. **(A)** Flow cytometric analysis of the F4/80 and CD11b expression in BMMs. Representative images of BMMs stained against **(B, C)** F4/80 and **(D, E)** CD11b. Representative images of cardiac fibroblasts stained against **(F, G)** vimentin, **(H, I)** CD31 (endothelial marker; negative control) and **(J, K)** desmin (smooth muscle marker; negative control). Nuclei were stained with DAPI **(C, E, G, I, K)** Magnification: 40x; scale bar: 50 µm. Data are representative of 3 independent experiments with similar results.

The purity of the BMM population was determined *via* flow cytometry analysis. 1x10^6^ cells were taken from the freshly harvested BMMs, processed and stained with the required antibodies according to the manufacturer’s protocol. The fluorescently labeled monoclonal antibodies (mAbs) that specifically recognize proteins expressed by macrophages were used for phenotypical characterization. The used two-color panel included two surface antigens, F4/80 (1:100, Miltenyi Biotec, Germany) and CD11b (1:100, Miltenyi Biotec, Germany). In this two-color immunofluorescence protocol, the samples were single stained with each antibody, and then stained using both antibodies. Data were acquired with a MACS-Quant device (Miltenyi Biotec, Germany) using the MACSQUANTIFY™ software (**Figure 1A**).

### Immunofluorescence

BMMs and murine cardiac fibroblasts were cultivated in 8-chamber slides (Sigma-Aldrich, Germany). Cells were fixed with 4% Histofix (Roth, Germany) and permeabilized with 0.2% Triton X-100 (Sigma-Aldrich). BMMs were stained against F4/80 (1:100, Abcam, UK) or CD11b (1:100, Abcam, UK) (**Figures 1B–E**). Fibroblasts were stained with antibodies against vimentin (1:100), CD31 (negative control for endothelial cells) (1:100), and desmin (negative control for smooth muscle cells) (1:100) (**Figures 1F–K**). The secondary antibodies anti-mouse FITC (1:100) (Dianova, Germany) or anti-mouse Cy3 (Dianova, Germany) were applied according to the manufacturer’s protocol. Nuclei were stained using DAPI (1:50000) (Sigma, Germany) and cells were mounted with Fluoromount G (Southern Biotech). Negative controls were performed by omitting the primary antibodies. Images were acquired using a BZ-9000E fluorescence microscope (Keyence, Germany). All evaluations were performed in a blinded manner.

### RNA Extraction and Quantitative Real-Time PCR

Total RNA from BMMs or murine cardiac fibroblasts was homogenized in RNA-Bee (Amsbio, UK). Quantitative real-time PCR was performed using the Brilliant SYBR Green qPCR master mix (Applied Biosystems, USA). The relative amount of target mRNA was determined using the comparative threshold (Ct) method as previously described (34). The mRNA content of target genes was normalized to the expression of hypoxanthine phosphoribosyl transferase (HPRT).

### Protein Extraction and Immunoblotting

BMMs were homogenized in a Laemmli buffer (253 mmol/l Tris/HCL pH 6.8, 8% SDS, 40% glycerin, 200 mmol/l Dithiothreitol, 0.4% bromophenol blue) (35). Proteins were quantified using the BCA Assay (Thermo Scientific Pierce Protein Biology, Germany). Equal amounts of total proteins were separated on SDS-polyacrylamide gels and transferred to a nitrocellulose membrane. The membranes were immunoblotted overnight with the following primary antibodies: NFκB (1:1,000, Santa Cruz, USA), ERα (1:100, Santa Cruz, USA), ERβ (1:200, Santa Cruz, USA), GPR30 (1:500, Santa Cruz, USA), ERK (1:1,000, Santa Cruz, USA) and p-ERK (1:2000, Santa Cruz, USA). Equal sample loading was confirmed by an analysis of actin (1:1,500, Santa Cruz, USA). Immunoreactive proteins were detected using ECL Plus (GE Healthcare, Buckinghamshire, UK) and quantified with ImageLab [version 5.2.1 build 11, Bio-Rad Laboratories (USA)].

### Total ROS Measurements

BMMs were loaded with 0.01 mmol/l DCF (2′,7′-dichlorodihydrofluorescein diacetate, succinimidyl ester) for total ROS measurement for 30 min. Subsequently, the cells were washed twice with PBS containing calcium-chloride (1 mmol/l) and lysed with a 0.5% TritonX-100 buffer. The fluorescence intensity was analyzed by excitation at 485 ± 10 nm and emission at 530 ± 10 nm using a ViktorX Multilable Plate reader and subsequently normalized to protein level.

### Statistical Analysis

The data are given as the mean ± SEM. The data were evaluated using the non-parametric test (Mann-Whitney test for two independent groups) or two-way ANOVA analysis. Statistical analyses were performed using GraphPad Prism 7 (GraphPad Software, San Diego, USA). Statistical significance was accepted when p < 0.05.

## Results

### Male and Female BMMs Express Estrogen Receptors

**Figure 2:**
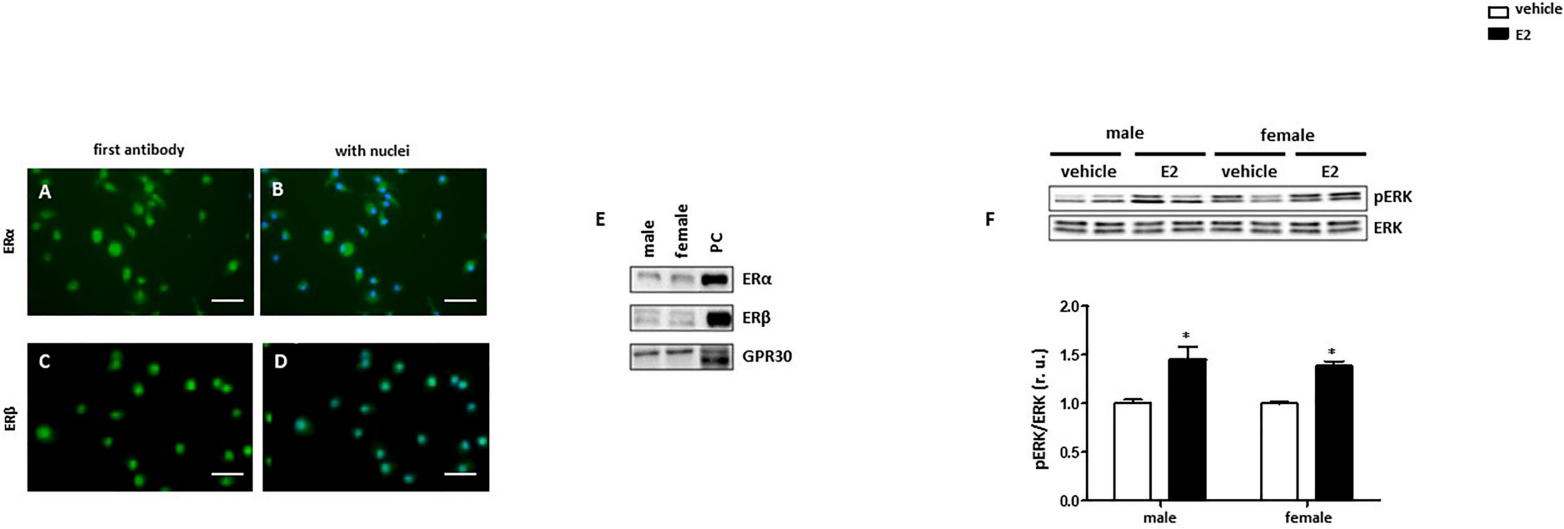
Male and female BMMs express active estrogen receptors. Representative images of BMMs cells stained against (green) **(A, B)** ERα and **(C, D)** ERβ. Nuclei were stained with DAPI (blue) **(B, D)**. Magnification: 40x, scale bar: 50 µm. Western blot analysis of **(E)** ERα, Erβ, and GPR30 and **(F)** pERK/ERK ratio in male and female BMMs with or without E2 treatment for 24 hrs. Data are representative of 3 independent experiments with similar results. Data are normalized to the male untreated group and expressed in relative units (r. u.). *p < 0.05, untreated *vs.* treated. PC = positive control (MCF7 lysate).

To investigate the effect of E2 on the polarization of macrophages, we first analyzed the expression of the estrogen receptors in male and female BMMs. All three estrogen receptors (ERα, ERβ and GPR30) are expressed in male and female BMMs (**Figures 2A–E**). Since the stimulation of ER activates ERK1/2 (36), the effects of E2 on ERK1/2 phosphorylation in BMMs were shown. 24h E2 treatment increased ERK phosphorylation in both sexes (**Figure 2F**).

### LPS Elicits Stronger Pro-Inflammatory Response in Male Than in Female BMMs

**Figure 3:**
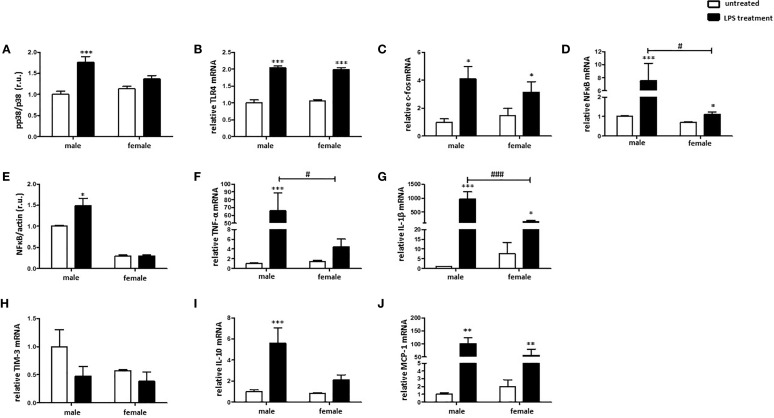
LPS elicits stronger pro-inflammatory response in male than in female BMMs. Expression analyses of **(A)** pp38/p38 ratio, **(B)** TLR4 mRNA, **(C)** c-fos mRNA, **(D)** NFκB mRNA, **(E)** NFκB protein, **(F)** TNF-α mRNA, **(G)** IL-1β mRNA, **(H)** TIM-3 mRNA, **(I)** IL-10 mRNA, and **(J)** MCP-1 mRNA performed with bone marrow macrophages lysates from male and female mice treated with 10 ng/ml LPS for 24 h. Data are shown as means ± SEM (n = 9; independent experiments with technical duplicates). Data are normalized to the male untreated group and expressed in relative units (r.u.). *p < 0.05, **p < 0.01, ***p < 0.001, untreated *vs.* treated; ^#^p < 0.05, ^###^p < 0.001, male *vs.* female.

To investigate sex differences in the M1 polarization of macrophages, male and female murine BMMs were treated with LPS. Mitogen-activated protein kinase p38 (p38) is a known downstream target of LPS and plays a crucial role in M1 macrophage polarization (12, 37). p38 was activated (indirectly highlighted by the phosphorylation rate) in male but not female BMMs after 24 h LPS treatment (**Figure 3A**). LPS treatment significantly increased c-fos and TLR4 expression at the RNA level in both sexes (**Figures 3B, C**). LPS treatment also significantly increased the NFκB mRNA in male and female macrophages, whereas the NFκB mRNA elevation in males was about four-fold higher than that in females (**Figure 3D**). Correspondingly, a significant increase of NFκB expression at the protein level was observed only in male macrophages after LPS treatment (**Figure 3E**). It is important to note that western blot assay also revealed a two-fold higher NFkB protein expression in males than females under basal conditions (**Figure 3E**), suggesting a pro-inflammatory phenotype in male BMMs under basal conditions. In accordance with these findings, TNF-α and IL-1β expression was about two times higher in male macrophages after LPS treatment (**Figures 3F, G**).

In contrast, LPS treatment downregulated the expression of the M2 marker, TIM-3, in male macrophages (**Figure 3H**). Both IL-10 as well as MCP-1, prominent M2 markers, were similarly upregulated in male and female macrophages after pro-inflammatory stimulus (**Figures 3I, J**).

### Male BMMs Show a More Prominent M2 Phenotype Than Females After IL4/IL13 Treatment

**Figure 4:**
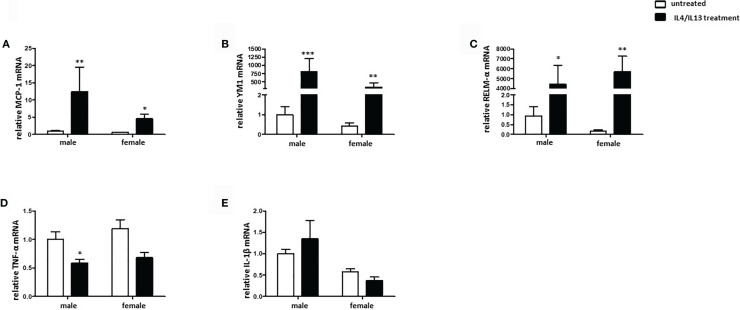
Male BMMs show a more prominent M2 phenotype compared with females. Real-time PCR analyses of **(A)** MCP-1, **(B)** YM1, **(C)** RELM-α, **(D)** TNF-α, and **(E)** IL-1β performed with bone macrophage lysates from male and female mice treated with 10 ng/ml IL-4 and 10 ng/ml IL-13 for 24h. Data are shown as means ± SEM (n = 9; independent experiments with technical duplicates). Data are normalized to the male untreated group. *p < 0.05, **p < 0.01, ***p < 0.001, untreated *vs.* treated.

To evaluate the sex differences in the polarization of M2 macrophages, the expression of specific markers was investigated. IL-4/IL-13 co-treatment significantly increased the MCP-1 mRNA expression in male and female BMMs (**Figure 4A**). In addition, YM1 mRNA expression was also upregulated in male and female M2 macrophages, whereas male macrophages showed about 2-fold stronger response than female cells (**Figure 4B**). The M2 marker RELM-α was also markedly upregulated in both male and female macrophages after IL-4/IL-13 co-treatment (**Figure 4C**). As might be expected, prominent pro-inflammatory markers, e. g., TNF-α was downregulated in male M2 macrophages (**Figures 4D**). Surprisingly, IL4/IL13 treatment had any effect on the expression of IL-1β neither in male nor in female macrophages (**Figure 4E**).

### TNF-α, but Not TGF-β, Activates a Pro-Fibrotic Phenotype in Mouse Cardiac Fibroblasts

**Figure 5:**
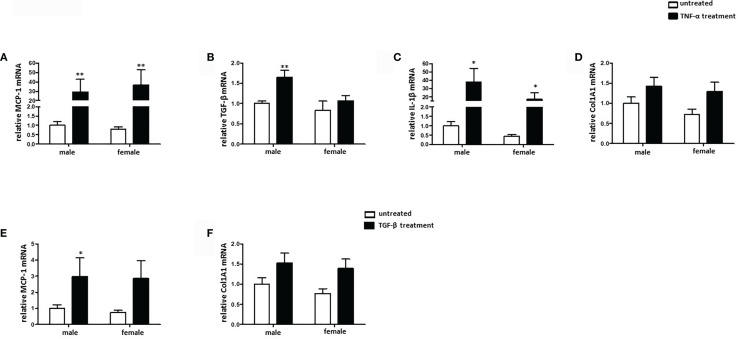
TNF-α, but not TGF-β, induces pro-fibrotic phenotype in mouse cardiac fibroblasts. Real-time PCR analyses of **(A)** MCP-1, **(B)** TGF-β, **(C)** IL-1β, and **(D)** Col1A1 performed with lysates from male and female mice cardiac fibroblasts treated with 20 ng/ml TNF-α for 24 h. Real-time PCR analyses of **(E)** MCP-1 and **(F)** Col1A1 performed with lysates from male and female mice cardiac fibroblasts treated with 10 ng/ml TGF-β for 24 h. Data are shown as means ± SEM (n = 6; independent experiments with technical duplicates). Data are normalized to the male untreated group. *p < 0.05, **p < 0.01, untreated *vs.* treated.

Sex differences in collagen expression and fibrosis formation are well established (31). To investigate the role of sex in fibroblast activation, cultured male and female mouse cardiac fibroblasts were treated with TNF-α or TGF-β. 24 h treatment with TNF-α significantly increased the mRNA expression of the pro-fibrotic markers MCP-1 and IL-1β, in male and female cardiac fibroblasts and the mRNA expression of TGF-β in male cardiac fibroblasts (**Figures 5A–C**), while the TGF-β expression was not increased in female cardiac fibroblasts after TNF-α treatment (**Figure 5B**). Furthermore, male fibroblasts showed a higher elevation of TGF-β and IL-1β (about 2.0-fold) under TNF-α treatment than female fibroblasts (**Figures 5B, C**). In contrast, 24 h treatment with TGF-β did not affect the expression of the pro-fibrotic marker MCP-1 in female fibroblasts, while it increased it in male cells (**Figure 5E**). Neither TNF-α, nor TGF-β affected the expression of Col1A1, a key marker involved in fibrosis formation, in cardiac fibroblasts (**Figures 5D, F**). In addition, MCP-1, TGF-β and IL-1β were increased in male cardiac fibroblasts after LPS treatment, while in female fibroblasts only MCP-1 and IL-1β were increased (data not shown).

### Pro-Inflammatory Macrophage Environment Promotes a Pro-Inflammatory and Pro-Fibrotic Fibroblast Phenotype

To investigate sex differences in the macrophage-fibroblast interaction, male and female cardiac fibroblasts were cultivated with the corresponding male or female conditioned medium from pro-inflammatory M1 BMMs.

**Figure 6:**
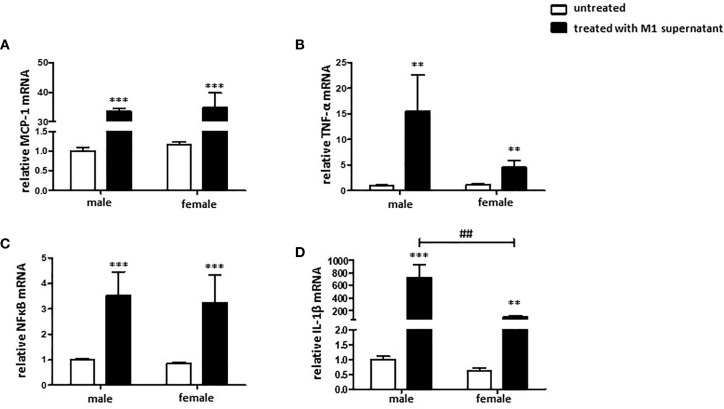
Pro-inflammatory environment promotes a pro-inflammatory and pro-fibrotic fibroblast phenotype. Real-time PCR analyses of **(A)** MCP-1, **(B)** TNF-α, **(C)** NFκB, and **(D)** IL-1β performed with lysates from male and female mice cardiac fibroblasts cultivated with conditioned medium from M1 polarized BMMs for 24 h. Data are shown as means ± SEM (n = 6; independent experiments with technical duplicates). Data are normalized to the male untreated group. **p < 0.01, ***p < 0.001, untreated *vs.* treated; ^##^p < 0.01, male *vs.* female.

Cultivation of male and female cardiac fibroblasts with a pro-inflammatory male or female M1 conditioned medium, respectively, increased the expression of MCP-1, TNF-α, NFκB, and IL-1β at the mRNA level in both sexes (**Figures 6A–D**). It is worth noting that the responses were more prominent in male than in female cells. Male fibroblasts showed in the TNF-α and IL-1β expression an about two-fold stronger response than female cells.

### Female BMMs Are More Protected Against Oxidative Stress After Pro-Inflammatory Stimulus

**Figure 7:**
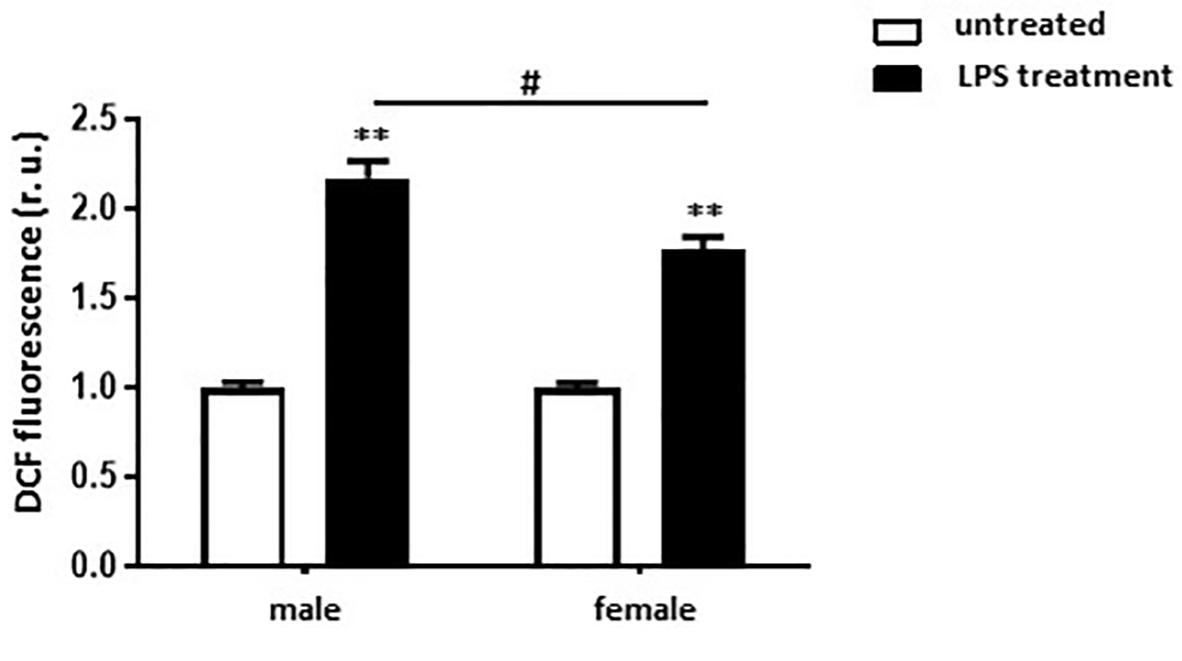
LPS treatment leads to stronger ROS formation in male than in female BMMs. Analysis of ROS formation (DCF fluorescence, r.u.) in untreated and M1 (10 ng/ml LPS for 24 h) male and female macrophages. Data are shown as means ± SEM (n = 6-7; independent experiments with technical duplicates). Data are normalized to the male untreated group. **p < 0.01, untreated *vs.* treated; ^#^p < 0.05, male *vs.* female.

To investigate the role of sex on ROS formation, total ROS level was measured in non-differentiated, as well as in M1 polarized male and female murine BMMs. M1 macrophages showed a two-fold higher ROS formation compared with untreated cells (**Figure 7**). Elevation of ROS was also more pronounced in male than in female M1 macrophages (**Figure 7**).

### E2 Treatment Promoted The Pro-Inflammatory Phenotype in Male BMMs

**Figure 8:**
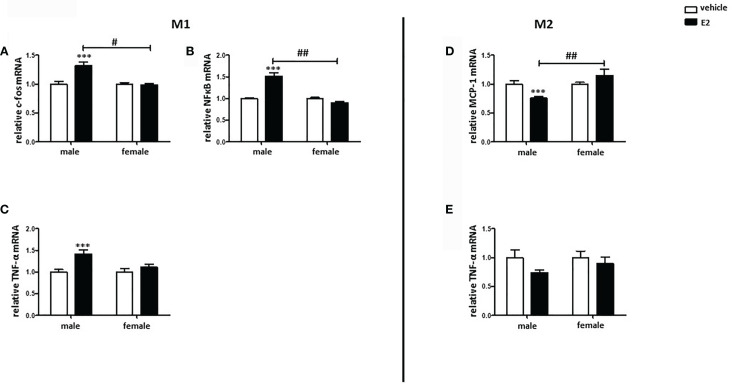
E2 treatment promoted the pro-inflammatory phenotype in male BMMs. Real-time PCR analyses of **(A)** c-fos, **(B)** NFκB, and **(C)** TNF-α in M1 macrophages, as well as **(D)** MCP-1 and **(E)** TNF-α in M2 macrophages with or without E2 treatment (10 nmol/l) for 24 h. Data are shown as means ± SEM (n=6; independent experiments with technical duplicates). Data are normalized to vehicle. ***p < 0.001, vehicle *vs.* E2 treated; ^#^p < 0.05, ^##^p < 0.01, male *vs.* female.

Both pro- and anti-inflammatory actions of E2 have been described (23, 24). To test the effects of E2, male and female M1 and M2 macrophages were post-treated with E2 for additional 24 h. The treatment significantly increased the expression of pro-inflammatory markers, e.g., c-fos, NFκB, and TNF-α, in male M1 macrophages, while it had no effects on female cells (**Figures 8A–C**). In M2 BMMs, E2 significantly decreased the expression of MCP-1 in male but not in female M2 macrophages (**Figure 8D**), while E2 had no effect on the expression of TNF-α (**Figure 8E**).

## Discussion

In the current study, we investigated sex-related alterations in the polarization of murine BMMs. The main findings are as follows: 1) Male BMMs show a stronger pro-inflammatory response to the LPS treatment than female BMMs; 2) Male BMMs show a more prominent M2 phenotype than females under IL4/IL13 treatment; 3) Treatment of cardiac fibroblasts with TNF-α or conditioned medium from M1 macrophages promotes a stronger pro-inflammatory and pro-fibrotic response in male cells; 4) E2 treatment promotes the pro-inflammatory and suppresses the anti-inflammatory phenotype in male BMMs.

### Sex-Dependent M1 and M2 Polarization

Activation of p38 is known as a downstream signaling of LPS and is important in M1 macrophage polarization signaling (12, 37). Our study revealed that p38 was activated *via* LPS in male but not female murine BMMs, suggesting that sex influences the activation of p38. In accordance with our results, p38 activation was higher in male than female myocardium after ischemia-reperfusion injury in rats, leading to a lower myocardial inflammatory response in females (38). Importantly, p38 activation promotes the expression of transcription factors such as NFκB or other pro-inflammatory mediators, e.g., TNF-α and IL-1β (39), suggesting that the male-specific p38 phosphorylation observed in this study may be translated into the stronger pro-inflammatory response.

Indeed, the majority of the pro-inflammatory markers show stronger responses to LPS treatment in male than in female macrophages. Particularly, the key pro-inflammatory transcription factor NFκB (12) was strongly upregulated in M1 male BMMs after 24 h LPS treatment. In addition, typical M1 signature cytokines, i.e., TNF-α and IL-1β, were also strongly upregulated in male cells. These data suggest that LPS treatment elicits a stronger pro-inflammatory response in male than in female BMMs. In line with our findings, the pro-inflammatory response increased in male hearts in a model of experimental autoimmune myocarditis, while female hearts showed less inflammation and an increased number of M2 macrophages, leading to a stronger induction of cardiac inflammation and cardiac dysfunction in male rats (40). It is interesting to note that MCP-1 and YM1 were significantly more upregulated in male than in female M2 macrophages polarized with IL4/IL13, however the expression of RELM-α was similar in male and female BMMs. This sex-dependent difference in the expression of anti-inflammatory markers in M2 BMMs might be explained by the difference in polarization of different M2 macrophage subspecies, e.g., M2a, M2b, M2c, and M2d, which have distinct functions and are activated by different stimuli (15, 30, 41). In contrast, pro-inflammatory M1 markers such as TNF-α were strongly downregulated in male M2 BMMs, suggesting that male macrophages are more susceptible to M2-macrophage polarization.

### Effects of M1 Polarization on ROS Formation

ROS formation plays a central role in many inflammatory diseases, as it is an important mediator of inflammation and cell injury (42). ROS formation was significantly increased after M1 polarization in both male and female BMMs, however the ROS level was significantly higher in male M1 macrophages than in female cells. In accordance with our results, Lagranha et al. reported that several mitochondrial-related sex differences are involved in the modulation of ROS homeostasis (43). Of note, sex hormones, especially E2, modulate mitochondrial ROS production (44, 45). Furthermore, it has been shown, in an atherosclerosis model using ovariectomized female mice, that E2 treatment decreased the expression of NADPH oxidase and the superoxide anion formation, while it increased the expression of two ROS-scavenging enzymes (Cu/ZnSOD and MnSOD), which suppose the E2 anti-oxidative effect (46).

### E2 Effects on BMM Polarization

E2 seems to play a crucial role in inflammatory processes (47, 48), and both pro-inflammatory as well as anti-inflammatory effects of E2 have been described (49, 50). In the present study, E2 treatment upregulated the expression of the pro-inflammatory markers, e.g., TNF-α, c-fos, and NFκB, in male M1 macrophages in a pro-inflammatory environment (LPS treatment), whereas it reduced the expression of the anti-inflammatory MCP-1 in male M2 macrophages exposed to an anti-inflammatory environment (IL4/IL13 treatment), suggesting that E2 promotes pro-inflammatory responses in male macrophages. In contrast, E2 had no effects on the expression of any markers investigated in female macrophages. In this regard, it was shown that E2 promotes sex-specific differences in the polarization of macrophages in an asthma animal model, as both male and female macrophages showed an increased expression of M2 genes induced by IL-4 after treatment with a specific ERα agonist, with stronger effects in females (51), thus profoundly impacting the immune system (52). In addition, Villa et al. proposed that E2 treatment decreases the M1 pro-inflammatory phenotype of macrophages, promoting the switch into M2c macrophages (53).

### Sex-Dependent Effects of Pro-Inflammatory Stimuli in Cardiac Fibroblasts

Activation of a pro-fibrotic program in cardiac fibroblasts may lead to pathological cardiac remodeling and heart failure (54). Activated fibroblasts express markers like MCP-1 and pro-fibrotic cytokines such as IL-1β (55–57). Moreover, Van Linthout describes TNF-α as crucial for fibroblast activation (21). In accordance with this, we demonstrated that TNF-α treatment significantly increased the expression of pro-fibrotic factors in a sex-independent manner. However, the TNF-α induced fibroblast activation was more prominent in male than in female fibroblasts and female fibroblasts expressed lower levels of pro-fibrotic factors such as TGF-β and IL-1β. Nevertheless, TNF-α did not change the expression of Col1A1 in neither male nor female fibroblasts. Col1A1, produced by fibroblasts *via* the TFG- β pathway, is fundamental for extracellular matrix synthesis and has been shown to play a key role in the development of diseases characterized by pathological fibrosis as well as the metastasis of various tumors (58, 59). Furthermore, TNF-α has been shown to decrease the Col1A1-expression in cultured fibroblasts (60), however our results did not support that finding.

In addition, TNF-α plays a crucial role in crosstalk between macrophages and fibroblasts (21). Depending on the macrophage subtype, macrophages can promote fibroblast activation or inhibition (41). Our study demonstrates an activation of cardiac fibroblasts with the pro-inflammatory supernatant of cultures of M1 macrophages, suggesting that a pro-inflammatory environment promotes a pro-inflammatory and pro-fibrotic phenotype in fibroblasts. Importantly, male fibroblasts showed more prominent effects from this treatment than female fibroblasts.

In addition, exposure to M1 supernatant strongly increased the ROS levels in male fibroblasts (unpublished data).

In conclusion, the present study revealed (i) a sex-dependent pro-inflammatory response to the M1 polarization stimuli in murine BMMs and (ii) a sex-dependent pro-inflammatory and pro-fibrotic response to the M1 macrophage environment in murine cardiac fibroblasts. The data suppose sex hormones and biological sex differences may play a pivotal role in the human immune system, which may dramatically affect cardiac inflammatory diseases, such as myocarditis.

### Limitations

We only investigated sex differences in macrophage polarization in C57/Bl6J mice. Since strain differences in the immune cell population have been reported (61, 62), we might consider strain differences in the macrophage polarization.

## Data Availability Statement

The original contributions presented in the study are included in the article. Further inquiries can be directed to the corresponding author.

## Ethics Statement

The animal study was reviewed and approved by the Animal Care Committee of the Senate of Berlin, Germany, approval number: T0333/08.

## Author Contributions

MB conceived the project, analyzed the data, prepared the figures, and wrote the main manuscript text. MN performed the molecular biological experiments and analyzed the data. CC performed the FACS analysis, characterization of ERs and ROS measurements, and analyzed the data. ME prepared figures and wrote the main manuscript text. NH performed molecular biological experiments and analyzed the data. AS performed the ROS measurements and analyzed the data. UM-W revised the manuscript. YL analyzed the data and wrote the main manuscript text. VR-Z generated research funds and coordinated the project. All authors commented on the manuscript.

## Funding

This work was supported by the DZHK (German Centre for Cardiovascular Research) and by the BMBF (German Ministry of Education and Research). We acknowledge support from the German Research Foundation (DFG) and the Open access Publication Fund of Charité – Universitätsmedizin Berlin.

## Conflict of Interest

The authors declare that the research was conducted in the absence of any commercial or financial relationships that could be construed as a potential conflict of interest.

## Publisher’s Note

All claims expressed in this article are solely those of the authors and do not necessarily represent those of their affiliated organizations, or those of the publisher, the editors and the reviewers. Any product that may be evaluated in this article, or claim that may be made by its manufacturer, is not guaranteed or endorsed by the publisher.
